# The relationship between ferroptosis and respiratory infectious diseases: a novel landscape for therapeutic approach

**DOI:** 10.3389/fimmu.2025.1550968

**Published:** 2025-03-18

**Authors:** Longyan Hong, Xiangyu Chen, Yiming Liu, Hao Liang, Yinghui Zhao, Pengbo Guo

**Affiliations:** ^1^ Department of Pathogen Biology, School of Clinical and Basic Medicine, Shandong First Medical University and Shandong Academy of Medical Sciences, Jinan, Shandong, China; ^2^ The First Affiliated Hospital of Shandong First Medical University, Shandong Provincial Qianfoshan Hospital, Jinan, Shandong, China; ^3^ Department of Health Inspection and Quarantine, School of Public Health, Cheeloo College of Medicine, Shandong University, Jinan, Shandong, China

**Keywords:** ferroptosis, role, respiratory infection, molecular mechanism, therapeutic approach

## Abstract

Respiratory infectious diseases, particularly those caused by respiratory viruses, have the potential to lead to global pandemics, thereby posing significant threats to public and human health. Historically, the primary treatment for respiratory bacterial infections has been antibiotic therapy, while severe cases of respiratory viral infections have predominantly been managed by controlling inflammatory cytokine storms. Ferroptosis is a novel form of programmed cell death that is distinct from apoptosis and autophagy. In recent years, Recent studies have demonstrated that ferroptosis plays a significant regulatory role in various respiratory infectious diseases, indicating that targeting ferroptosis may represent a novel approach for the treatment of these conditions. This article summarized the toxic mechanisms underlying ferroptosis, its relationship with respiratory infectious diseases, the mechanisms of action, and current treatment strategies. Particular attentions were given to the interplay between ferroptosis and Mycobacterium tuberculosis, Epstein-Barr virus, severe acute respiratory syndrome coronavirus-2, Pseudomonas aeruginosa, dengue virus, influenza virus and herpes simplex virus type1infection. A deeper understanding of the regulatory mechanisms of ferroptosis in respiratory infections will not only advance our knowledge of infection-related pathophysiology but also provide a theoretical foundation for the development of novel therapeutic strategies. Targeting ferroptosis pathways represents a promising therapeutic approach for respiratory infections, with significant clinical and translational implications.

## 1 Introduction

In 2012, Brent Stockwell, Scott Dixon, and colleagues introduced the concept of iron-dependent regulatory cell death, termed ferroptosis (1). This oxidative form of cell death is distinct from apoptosis, non-regulatory necrosis, and necroptosis (regulated necrosis) (2). Ferroptosis is primarily triggered by the accumulation of lethal lipid hydroperoxides within cellular membranes, particularly in the endoplasmic reticulum (ER), due to the failure of protective mechanisms that prevent excessive lipid peroxidation (3). Respiratory diseases pose a significant global health burden, ranking as the third leading cause of mortality in urban areas and the leading cause of death in rural regions (4, 5). Historically, the primary treatment for bacterial infections has been antibiotic therapy. However, the emergence of multiple drug-resistant strains has posed significant challenges to the effectiveness of such treatments (6). In contrast, there are limited specific antiviral medications available; most existing drugs primarily function by inhibiting viral replication or transmission within the host (7). Consequently, it is essential to elucidate the mechanisms underlying respiratory bacterial and viral infectious diseases in order to identify new insights and develop innovative therapeutic strategies. The discovery of ferroptosis has significant implications for respiratory diseases caused by certain viruses and bacteria, offering new avenues for treatment (8), particularly in targeting host-pathogen interactions to mitigate disease severity and improve clinical outcomes.

## 2 The relationship between ferroptosis and respiratory infection

### 2.1 Mycobacterium tuberculosis

**Figure 1:**
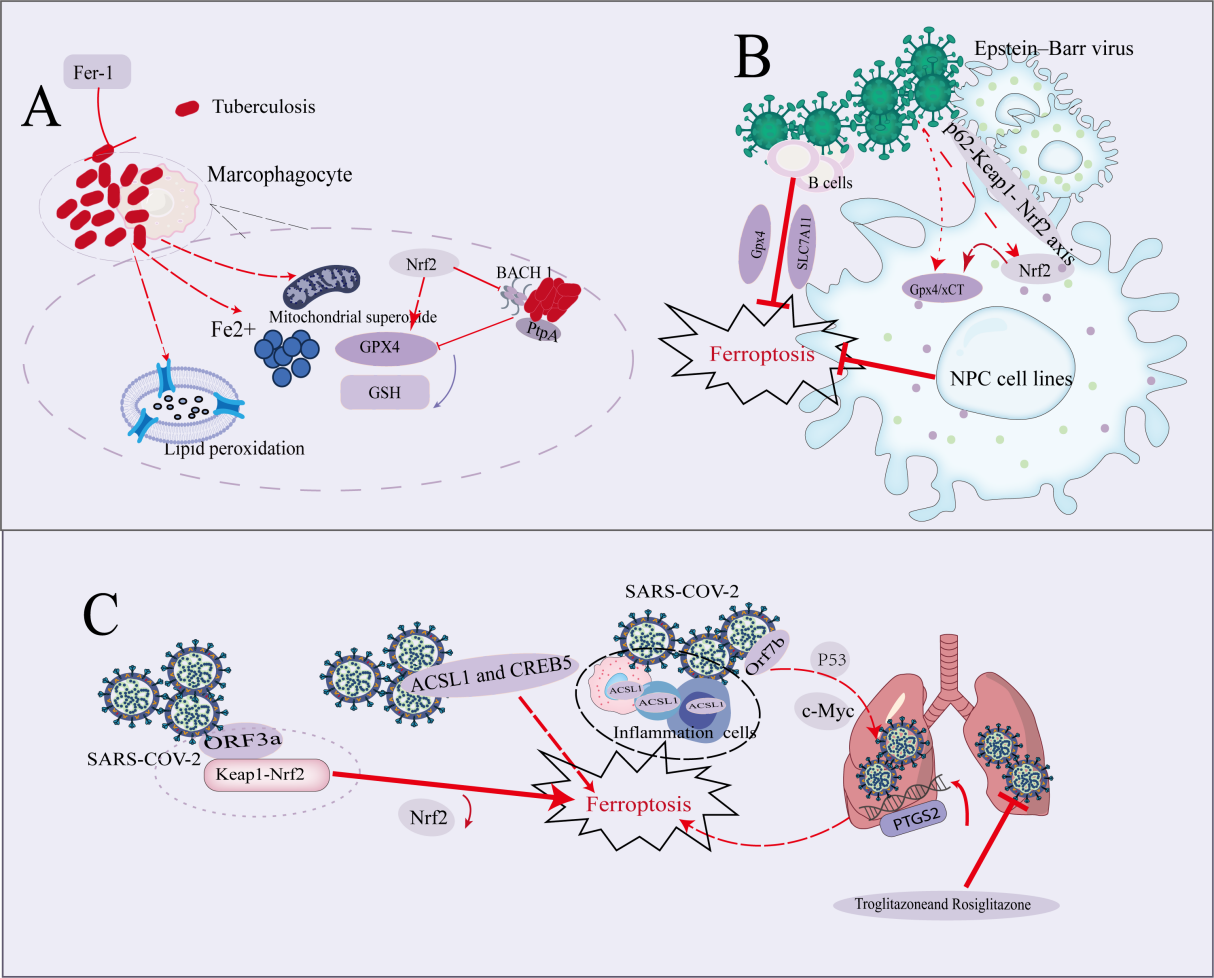
Overview Diagram of Ferroptosis Mechanism in Mycobacterium tuberculosis, EB Virus, and SARS-CoV-2 Infections. **(A)** The necrosis of macrophages triggered by *M. tuberculosis* is closely linked to elevated levels of intracellular iron, increased mitochondrial superoxide, and enhanced lipid peroxidation. The use of iron chelators or inhibitors of lipid peroxidation effectively averted *M. tuberculosis*-induced macrophage necrosis. The nuclear factor (Nrf2) played a role in inhibiting the expression of Bach1 and regulating Gpx4 to mitigate ferroptosis. Additionally, the effector molecule PtpA secreted by *M. tuberculosis* promoted ferroptosis, thereby increasing its pathogenicity and ability to spread. **(B)** This panel outlines the molecular mechanisms through which EBV infects B cells and nasopharyngeal carcinoma (NPC) cell lines. EBV infection makes B cells reliant on the activity of SLC7A11 and Gpx4 to resist ferroptosis. In NPC cell lines, EBV infection activates the p62-Keap1-NRF2 pathway, leading to enhanced Nrf2 activity. This activation further upregulates downstream target genes, such as Gpx4 and xCT, which play a critical role in reducing lipid peroxidation and inhibiting the accumulation of lipid ROS, thereby preventing ferroptosis. **(C)** The accessory protein Orf7b of SARS-CoV-2 promotes ferroptosis through the c-Myc signaling pathway, leading to lung damage. The expression of ferroptosis-related genes ACSL1 and CREB5 in monocytes may further promote ferroptosis by regulating the inflammatory response. In lung cells infected with SARS-CoV-2, the mRNA levels of the ferroptosis marker PTGS2 are significantly increased. Additionally, ORF3a is considered a positive regulator of ferroptosis; it binds to Keap1, enhancing Keap1 stability, promoting the interaction between Keap1 and Nrf2, and thereby accelerating the degradation of Nrf2, which in turn facilitates ferroptosis.

*M. tuberculosis* infection causes tuberculosis (TB), which ranks among the top ten causes of death worldwide (9). Prior to the COVID-19 pandemic, it was the leading cause of mortality attributable to a single infectious pathogen (10). TB is characterized as a chronic respiratory disease, manifesting a range of symptoms, including cough, hemoptysis, dyspnea, and chest pain. We summarized the role and molecular mechanisms of ferroptosis-related molecules in *M. tuberculosis* infection to provide novel insights and research directions for the treatment of this infection (**Figure 1A**).

#### 2.1.1 The role of ferroptosis in cell death and tissue necrosis induced by *M. tuberculosis*

*M. tuberculosis*-induced necrotic cell death was associated with increased levels of intracellular iron and mitochondrial superoxide, as well as lipid peroxidation (11). Using an *in vitro* model of *M. tuberculosis*-induced macrophage death, researchers observed that necrosis exhibited classic characteristics associated with ferroptosis. The necrotic cell death of macrophages infected with *M. tuberculosis* was linked to decreased expression levels of GSH and Gpx4 (12, 13) (**Figure 1A**).

Iron chelation or inhibition of lipid peroxidation could prevent macrophage necrosis induced by *M. tuberculosis*. Fer-1 is widely recognized as a potent inhibitor of ferroptosis due to its highly effective inhibition of lipid peroxidation, partly attributed to its antioxidant activity (1). Treatment with Fer-1 significantly reduced lung weight and calculated relative lung mass in infected mice (**Figure 1A**). Histological examination confirmed that this inhibition of lipid peroxidation correlates with a reduction in granulomatous inflammation.

#### 2.1.2 Gpx4 regulated cell necrosis and host resistance in *M. tuberculosis* infection

By comparing tuberculosis patients with healthy individuals, it was observed a decrease in the levels of Gpx4/GSH and an increase in lipid peroxidation among TB patients (10, 14).One potential strategy is to target upstream signaling components that positively regulate Gpx4 expression and/or activity in glutathione metabolism. A key component in this process was nuclear factor erythroid 2-related factor 2 (Nrf2), which not only regulated Gpx4 expression but also influenced various other antioxidant molecules (**Figure 1A**). However, this approach requires extensive clinical trials for further validation (10, 15–17).

In addition, some antioxidants, including vitamin E, were shown to inhibit ferroptosis, and studies indicated that adjunctive use of vitamin E with conventional antibiotic therapy improved TB treatment outcomes (13, 18). Furthermore, supplementing selenium was shown to enhance Gpx4 expression and activity, thereby inhibiting ferroptosis (12). Notably, selenium, as a nutritional supplement, was demonstrated to improve TB treatment outcomes (13).

#### 2.1.3 Protein tyrosine phosphatase A promoted pathogenicity and transmission dependent on ferrous breakdown

PtpA is an effector secreted by *M. tuberculosis*. PtpA triggered ferroptosis, thereby enhancing the pathogenicity and transmission of *M. tuberculosis*. It interacted with the host RanGDP through its Cys 11 site to enter the host cell nucleus. Once inside, nuclear PtpA promoted the asymmetric dimethylation of histone H3 at arginine 2 (H3 R2 me2a) by targeting protein arginine methyltransferase 6 (PRMT 6), resulting in the inhibition of Gpx4 expression and ultimately inducing ferroptosis, which facilitates *M. tuberculosis* pathogenicity and spread (**Figure 1A**) (19–21).

#### 2.1.4 BACH 1 promoted the enrichment of genes related to the inhibition of iron cell proliferation, leading to tissue necrosis and susceptibility to *M. tuberculosis*

The exacerbation of lipid peroxidation is a consequence of uncontrolled oxidative stress, which is linked to iron deposition and necrotic cell death (22–24).Bach 1 is a transcription factor that inhibits a variety of antioxidant genes. Notably, BACH 1 mRNA expression is increased in patients who develop active TB following *M. tuberculosis* infection. Importantly, it was observed that iron sepsis-related genes associated with antioxidant properties were upregulated in various bone marrow cell populations in *M. tuberculosis*-infected Bach 1 −/− mice, suggesting a role for Bach 1 in regulating *M. tuberculosis*-induced necrosis *in vivo*. Under conditions of Bach 1 deficiency, complete ablation of cell necrosis was not observed in either *in vivo* or *in vitro* settings, suggesting that Bach 1 deficiency and its role in cell necrosis coexist.

Nrf2 is the primary upstream regulator of the host antioxidant response (including GSH metabolism), and inhibiting its repressive factor Bach might provide an alternative strategy to enhance the expression of enzymes and their cofactors, which were crucial for mitigating lipid peroxidation-mediated tissue death (25). Discovering and developing other biologically acceptable Bach 1 inhibitors represented an effective strategy for addressing various diseases, particularly those where lipid peroxidation-mediated cell necrosis is recognized as a significant mechanism of pathogenesis (1, 23, 24, 26).

### 2.2 Epstein–barr virus

EBV is a double-stranded DNA virus that establishes lifelong latency in the host following primary infection (27). EBV infection has been closely associated with various B-cell malignancies (28–31). Among these, the malignancy most strongly associated with EBV infection has been undifferentiated nasopharyngeal carcinoma (NPC) (32). Although chemotherapy and radiotherapy are the primary treatment modalities for NPC, significant challenges remained due to chemotherapy resistance. Nevertheless, there had been considerable advancements in understanding the pathogenesis and exploring treatment options for the role of ferroptosis EBV infection (33).

#### 2.2.1 EBV infection modulates B cell resistance to ferroptosis

EBV activated lipid metabolism, transforming B cells into immortalized lymphoblastic cell lines (LCLs), which served as a model for post-transplant lymphoproliferative disorders and promoted lipid peroxidation in primary B cells. Anti-EBV therapy remains a significant unmet medical need, especially for immunocompromised patients. Although antiviral drugs approved for other herpesviruses have been tested for EBV-associated diseases, their results have been disappointing (31). Studies showed that dipyridamole (DIP), a drug with good safety and broad pharmacological properties, effectively inhibited EBV reactivation in B cell lines (34). Additionally, research found that different stages of EBV transformation generate varying levels of lipid-derived reactive oxygen species (ROS) byproducts. In the early stages of infection (Burkitt-like hyperproliferation phase), EBV induces lipid metabolism and ROS production, making B cells dependent on cystine import mediated by SLC7A11 and the activity of Gpx4 to resist ferroptosis (**Figure 1B**). Blocking these pathways, such as using erastin to inhibit SLC7A11 or ML-210 to inhibit Gpx4, significantly induces cell death (35). This phenomenon suggested that ferroptosis could be a potential therapeutic strategy for preventing or treating certain EBV-associated lymphomas (35).

#### 2.2.2 EBV infection-induced-Gpx4 promoted chemotherapy resistance in NPC

By suppressing Gpx4 expression, EBV promoted the escape of ferroptosis and the restoration of redox homeostasis, thereby contributing to chemotherapy resistance in NPC. EBV infection led to the upregulation of p62, which subsequently activated Nrf2 in NPC cell lines through the p62-Keap1- Nrf2 axis. This activation resulted in the upregulation of downstream effectors xCT and Gpx4, effectively reducing lipid ROS accumulation in tumor cells and protecting them from ferroptosis (**Figure 1B**). Gene knockout of Gpx4 or treatment with low-dose Gpx4-targeted inhibitors could significantly diminish chemotherapy resistance in EBV-positive NPC cell lines, presenting a potential therapeutic target for treating chemotherapy-resistant tumors (35–37).

### 2.3 Severe acute respiratory syndrome coronavirus-2

SARS-CoV-2 was a novel coronavirus identified as the causative agent of the global epidemic of coronavirus disease 2019 (COVID-19) (38–41). This virus belonged to the coronavirus family and was a highly transmissible airborne infectious pathogen (41). COVID-19 primarily presented as a respiratory disease that manifested as acute upper and/or lower respiratory syndrome of varying severity. The most common symptoms included fever, cough, acute lung injury, septic shock, and acute respiratory distress syndrome (ARDS) (42, 43). Additionally, various other symptoms had been reported in COVID-19 patients, such as loss of taste or smell, headache, muscle or body pain, nausea or vomiting, and diarrhea (44, 45). (The relationship between SARS-CoV-2 infection and the ferroptosis mechanism is illustrated in **Figure 1C** as a schematic diagram).

#### 2.3.1 Identification of iron metabolism related biomarkers in SARS-CoV-2 induced ischemic stroke using single-cell RNA sequencing and multiple bioinformatics methods

The seven main cell types identified in COVID-19 patients, which are affected peripherally by SARS-CoV-2, include monocytes, NK cells, platelets, CD34+ pre-B cells, T cells, B cells, and HSC-G-CSF cells. The ferroptosis-related genes ACSL1 and CREB5 were predominantly expressed in monocytes, HSC-G-CSF cells, and CD34+ pre-B cells (46, 47). The esterification of acyl-CoA synthase long-chain member 4 (ACSL4) allowed polyunsaturated fatty acids (PUFAs) to disrupt cell membranes during the execution phase (48). Recently, ACSL1 had been identified as a promoter of ferroptosis (47) (**Figure 1C**), withACSL1-induced gallic acid (ESA) triggered ferroptosis. In contrast to typical ferroptosis inducers such as the Gpx4 inhibitor ML160 and the FSP1 inhibitor iFSP1, the ACSL1-dependent ferroptosis induced by ESA shows significant differences (49). The high expression of five ferroptosis-related genes (such as ACSL1 and CREB5) in monocytes of COVID-19 patients may affect the inflammatory response, which could further influence the development of stroke, particularly in strokes triggered by COVID-19 infection (50, 51).Targeted ferroptosis may serve as a potential therapeutic approach for managing excessive inflammation induced by coronavirus infection (52).

#### 2.3.2 SARS-CoV-2 helper protein Orf 7b induced lung injury through ferroptosis

Diffuse alveolar injury (DAD) was a significant pathological feature observed in autopsy reports of COVID-19 patients, characterized by extensive cell death and lung injury indicative of disease exposure (53–56). Previous study investigated Orf 7b promoted ferroptosis through the upregulation of c-Myc. Researches had indicated that c-Myc could inhibit ferroptosis, and it was established that P53 suppressed c-Myc via a histone deacetylation mechanism (57–59). Thus, Orf 7b-mediated P53 inhibition might represent a potential mechanism for c-Myc activation, providing a foundation for future investigations (**Figure 1C**).

#### 2.3.3 SARS-CoV-2 ORF3a sensitized cells to ferroptosis via Keap 1-Nrf2 axis

PTGS2 is a biomarker associated with ferroptosis. In lung cells infected with SARS-CoV-2, the mRNA level of PTGS2 significantly increased (60)(**Figure 1C**). In contrast, the transcription levels of ferroptosis regulatory factors—such as solute carrier family 7member 11 (SLC7A11), ferritin light chain (FTL), ferritin heavy chain 1 (FTH1), NAD(P)H quinone dehydrogenase 1 (NQO1), heme oxygenase-1 (HO-1), and Gpx4—in lung tissue significantly decreased following SARS-CoV-2 infection. These findings indicated a substantial association between SARS-CoV-2 infection and ferroptosis. Among the seven accessory proteins, cells overexpressing SARS-CoV-2 ORF3a exhibited the highest levels of lipid ROS induced by RSL3. Both erastin and RSL3 promoted significant cell death and increased lipid ROS levels. Evaluating whether ORF3a expression renders cells sensitive to erastin and RSL3-induced ferroptosis revealed that the inhibition of cell growth and lipid ROS formation induced by erastin and RSL3 increased significantly in a dose-dependent manner. This effect could be completely reversed by iron-induced apoptosis inhibitors (e.g., DFO, Fer-1), but not by apoptosis or necrosis inhibitors. These results indicated that SARS-CoV-2 ORF3a acted as a positive regulator of ferroptosis in cells.

Research had identified that the Nrf2- ARE pathway regulated ferroptosis mediated by ORF3a, with the iron-inhibiting gene driven by Nrf2 being suppressed in SARS-CoV-2 infected cells, making them more susceptible to ferroptosis. The Keap1- Nrf2 axis was known to prevent ferroptosis. ORF3a directly bind to Keap1, enhancing its stability and promoting the interaction between Keap1 and Nrf2. This interaction facilitated the degradation of Nrf2, thereby alleviating the inhibition of ferroptosis (**Figure 1C**). Notably, knockdown of Keap1 partially rescued lipid ROS levels in cells overexpressing ORF3a, thus continuing to prevent iron-induced apoptosis (61). Two types of ferroptosis inhibitors, troglitazone and rosiglitazone, had been shown to reduce viral production of coronaviruses, including SARS-CoV-2 (62) (**Figure 1C**).

### 2.4 Pseudomonas aeruginosa

*P. aeruginosa* is an obligate aerobic bacterium capable of infecting any tissue or organ in the human body, particularly when the immune system is compromised. Following infection of the respiratory tract, pneumonia may develop, predominantly in patients with impaired pulmonary immune function, such as those with cystic fibrosis or those who have undergone tracheal intubation. In severe cases, the infection could lead to complications such as heart failure and emphysema. Currently, pharmacological therapy is the primary treatment approach in clinical practice, often requiring prolonged duration (63–66). Recent studies had demonstrated that ferroptosis played a crucial regulatory role in *P. aeruginosa* infections. These findings offered novel insights for the prevention and treatment of *P. aeruginosa* infections in the future (67).

#### 2.4.1 Lipid peroxidation induced by *P. aeruginosa* leaded to ferroptosis

**Figure 2:**
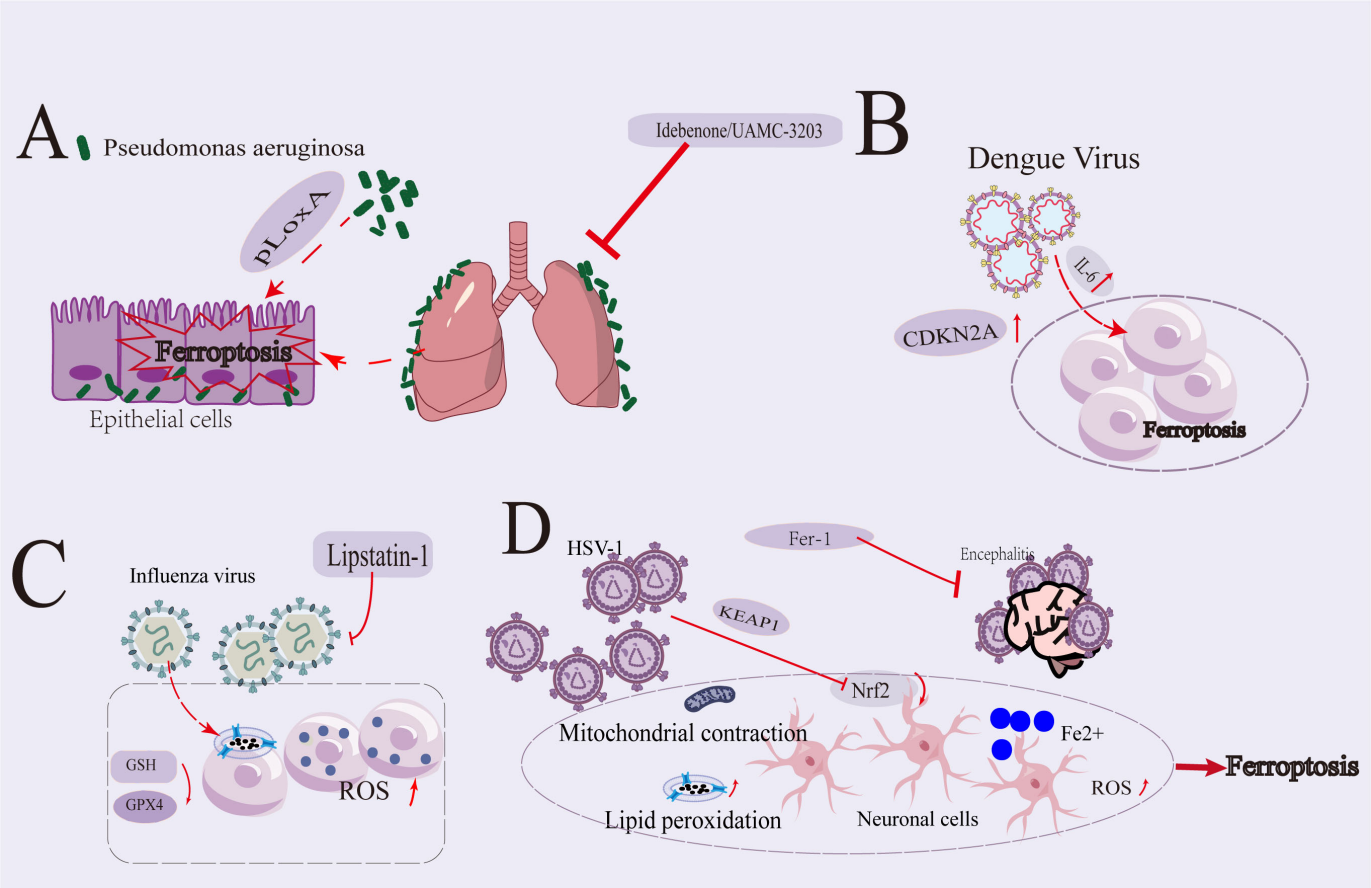
Overview Diagram of the Role of Ferroptosis Mechanism in Pseudomonas aeruginosa, Dengue Virus, Influenza Virus, and Herpes Simplex Virus 1 Infections. **(A)** *P. aeruginosa* triggered ferroptosis in human bronchial epithelial cells through the secretion of lipoxygenase (pLoxA). Inhibitors of iron-mediated cell death significantly mitigated cellular apoptosis in the lungs affected by cystic fibrosis and in inflammatory responses associated with bacterial pneumonia. **(B)** During DENV infection, IL-6, functioning as a pro-inflammatory cytokine, facilitated ferroptosis through the induction of ROS-mediated lipid peroxidation and the perturbation of iron homeostasis. Furthermore, there was an upregulation of CDKN2A expression in HepG2 cell lines infected with DENV. **(C)** The influenza virus impeded its own replication by increasing the accumulation of lipid peroxidation metabolites and lipid ROS, along with the depletion of intracellular GSH and the downregulation of Gpx4. Lipstatin-1 had the capacity to inhibit the intracellular propagation of the virus. **(D)** HSV-1 precipitated ferroptosis in both cellular and organismal models. HSV-1 augmented the KEAP1-mediated proteasomal degradation of Nrf2, thereby facilitating ferroptosis. The ferroptosis triggered by HSV-1 infection was typified by iron accumulation, ROS buildup, GSH depletion, lipid peroxidation, and mitochondrial shrinkage. Additionally, ferroptosis induced by HSV-1 plays a significant role in the development of viral encephalitis in mice. Inhibition of ferroptosis by Fer-1 alleviates this process.

Dar et al. reported the expression of lipoxygenase (pLoxA) in *P. aeruginosa*, the host’s arachidonic acid phosphatidylethanolamine was oxidized, triggering ferroptosis in human bronchial epithelial cells (**Figure 2A**). Idebenone, an effective antioxidant compound and inhibitor of TMEM 16A, had been approved for use in treating Duchenne muscular dystrophy and Leber hereditary optic neuropathy (68–71). Furthermore, it could mitigate lipid peroxidation and cell death in the lungs of cystic fibrosis patients. Additionally, the direct inhibition of *P. aeruginosa*-induced cell death in CF lungs and other forms of bacterial pneumonia by a new generation of ferroptosis (UAMC-3203) inhibitors might significantly reduce lung inflammation (72) (**Figure 2A**).

#### 2.4.2 *P. aeruginosa* enhanced radiation damage by inducing ferroptosis

It was found that *P. aeruginosa* significantly exacerbated the damage caused by TBI, further confirming that the pLoxA-rich vesicles secreted by *P. aeruginosa* triggered ferroptosis in epithelial cells (66). Additionally, selectively targeting pLoxA might represent a promising therapeutic strategy aimed at reducing the high mortality rate associated with TBI combined with *P. aeruginosa* infection (73, 74).

### 2.5 Dengue virus

Dengue fever (DF), a global arthropod-borne viral disease, is caused by infection with the DENV, primarily transmitted through mosquito bites (75, 76). DENV infection induced a characteristic pathology in humans, manifesting as vascular system dysfunction. However, the pathogenesis of DENV remained incompletely elucidated, as many viruses exploited host responses to facilitate their own replication (77). Recent studies had indicated a potential relationship between iron dysregulation and DENV infection (78). (**Figure 2B** illustrates the potential relationship between dengue virus and ferroptosis).

Li et al. identified and validated nine key genes associated with iron metabolism and DENV infection (HSPA5, CAV1, HRAS, PTGS2, JUN, IL6, ATF3, XBP1, and CDKN2A). IL-6, PTGS2, ATF3, and XBP1 were categorized as markers of ferroptosis and might signify the presence of ferroptosis during DENV infection. JUN is regarded as a suppressor gene of ferroptosis, while HRAS, NRAS, ATF3, and CDKN2A were considered driver genes that might promote ferroptosis. DENV infection had been shown to increase IL-6 production, which was associated with the pathogenesis of severe DENV disease. Reports indicated that IL-6, a pro-inflammatory cytokine, promoted ferroptosis by inducing cellular ROS-dependent lipid peroxidation and disrupting iron homeostasis (**Figure 2B**). These findings suggested that IL-6 played a crucial role in the pathogenesis of DENV infection by regulating ferroptosis (79–83).

CDKN2A, also known as ARF, stimulated ROS-induced ferroptosis in a p53-independent manner. Previous researches suggested that IL-6, ATF3, XBP1, and CDKN2A were closely associated with iron dysregulation and viral infection, providing a foundational basis for our hypothesis that these genes may be involved in the pathogenesis of DENV infection through the regulation of host cell iron homeostasis. The potential relationship between ferroptosis and DENV infection was elucidated based on a microarray gene expression dataset from a public database using bioinformatics analysis of an exojunction cell culture model (78, 84, 85). This finding provided a basis for further research on the specific regulatory role of ferroptosis in the pathogenesis of dengue fever and laid the foundation for the development of new therapeutic strategies.

### 2.6 Influenza virus

Influenza virus is classified into three types: A, B, and C. The acute respiratory infection caused by the influenza virus is known as influenza. Influenza A and B viruses frequently led to seasonal epidemics, resulting in localized outbreaks, while influenza A viruses have the potential to cause a global pandemic. Recent studies had indicated a significant relationship between influenza virus infection and ferroptosis (86–89).

Influenza virus infection led to excessive accumulation of intracellular ROS, and the replication of influenza A virus is influenced by the REDOX(Reduction-Oxidation) state of the host cell, including GSH level (90). Increasing evidence suggested a correlation between the intracellular replication of influenza virus and cell iron level (91). The influenza virus induced ferroptosis by accumulating lipid ROS, depleting GSH, and downregulating Gpx4 expression (**Figure 2C**). Hemagglutinin (HA) induced ferroptosis via ferritinophagy, leading to lipid peroxidation and impairing the MAVS (mitochondrial antiviral signaling)-mediated type I interferon response, ultimately promoting viral replication (92). Additionally, Lipstatin-1 inhibited the intracellular replication of the virus, indicating that the replication of influenza viruses is reliant on cellular iron levels (14, 89–91).

### 2.7 Herpes simplex virus type 1

HSV-1 is an enveloped double-stranded DNA virus that belongs to the genus Herpes Simplex virus within the Herpesviridae family. It is a ubiquitous and highly contagious pathogen that primarily infects humans. HSV-1 primarily causes contact infections through the oral or ocular mucosa, can infiltrate the nervous system, and establishes latent infections via complex immune evasion mechanisms (14, 93, 94).

The study conducted by Xu et al. demonstrated that HSV-1 could induce ferroptosis in both *in vitro* and *in vivo*. Specifically, HSV-1 enhanced KEAP1-dependent Nrf2 degradation, which contributed to ferroptosis. (**Figure 2D** illustrates the potential relationship between HSV-1 infection and ferroptosis.) Furthermore, exogenous GSH inhibited HSV-1 replication by interfering with the later stages of the virus lifecycle, without affecting cellular metabolism (95).

Findings suggested that HSV-1-induced ferroptosis was crucial to the neuro-pathogenesis of the virus. Evidence showed that HSV-1 infection led to ferroptosis characterized by iron overload, ROS, GSH depletion, lipid peroxidation, and mitochondrial contraction in cultured astrocytes, microglial cells, and mouse brains (**Figure 2D**). Additionally, HSV-1 infection promoted the ubiquitination and degradation of KEAP1-dependent Nrf2, resulting in a significant decrease in the expression levels of downstream anti-iron deposition genes, thereby disrupting cellular redox homeostasis and promoting iron accumulation (1, 96).

The interplay between HSV-1 infection and ferroptosis provided new insights into the physiological impact of ferroptosis on the pathogenesis of HSV-1 infection and encephalitis (**Figure 2D**). Furthermore, the inhibition of ferroptosis showed promise as a therapeutic strategy against HSV-1-induced encephalitis, highlighting its potential as an effective immunotherapy approach for treating HSV-1 infections and associated encephalitis (97).

## 3 Conclusion

Ferroptosis is a type of regulated cell death distinct from apoptosis and necrosis. It is associated with amino acid metabolism, iron metabolism, lipid metabolism, and mitochondrial activity (2). Ferroptosis is implicated in processes such as inflammation, oxidative stress, and lipid peroxidation. Additionally, it is associated with vascular diseases, including Alzheimer’s disease, stroke, and ischemia-reperfusion injury (98).

Numerous studies had demonstrated a close relationship between ferroptosis and respiratory pathogenic microbial infection (78, 99–105). Tuberculosis, caused by infection with *M. tuberculosis*, remains a significant global public health issue (106). The current lack of safe and effective adult tuberculosis vaccines, the need for prolonged and adequate antibiotic treatment, the increase in global population, and the concentration of populations in underdeveloped areas have all contributed to the increased challenges in the prevention and treatment of *M. tuberculosis* infection (107, 108). This challenge has spurred significant interest in developing new strategies to target *M. tuberculosis* infection. EBV is the first identified virus associated with cancer. However, the precise mechanisms by which EBV influences the progression of malignancies in related tumors remain unclear, posing challenges for the prevention and treatment of EBV-associated cancers (109, 110). SARS-CoV-2 caused a global pandemic at the end of 2019 (111). Despite the emergence and administration of multiple vaccines against SARS-CoV-2, infections continue to occur. Cytokine storms are a significant factor contributing to mortality in severe cases of SARS-CoV-2 infection (112, 113). The emergence of multidrug-resistant strains of *P. aeruginosa* infections, particularly among ICU patients, presents significant challenges to treatment (114). Dengue fever is highly prevalent in tropical and subtropical regions, particularly in Africa, where mosquito-borne fatalities are common (115). The influenza A virus has caused numerous global pandemics, resulting in tens of millions of deaths. Despite the use of vaccines against certain strains of the virus, its multiple serotypes and rapid mutation rate continue to pose significant challenges for prevention, control, and treatment (116, 117). This remains a particularly pressing issue in many countries, especially Japan. HSV-1 spreads rapidly and has a high prevalence among humans. In addition to causing oral herpes, it can lead to severe diseases affecting the central nervous system and circulatory system, which are significant factors in mortality (118, 119).Consequently, the investigation of ferroptosis inhibitors offered new avenues for the treatment of these respiratory infectious diseases, which was summarized in **Table 1**.

**Table 1:** Ferroptosis mechanisms and therapeutic strategies in respiratory infectious diseases.

Pathogen	Mechanism of Ferroptosis	Functionality Importance	Treatment Methods
Mycobacterium tuberculosis *(M. tuberculosis*)	*M. tuberculosis* induces macrophage necrotic cell death by increased intracellular iron, mitochondrial superoxide, lipid peroxidation, and regulation of Gpx4/GSH levels.	Ferroptosis enhances pathogenicity, promotes necrosis, and facilitates bacterial spread.	Use iron chelators (such as deferoxamine) and ferroptosis inhibitors (such as Fer-1) to prevent tissue necrosis, as well as develop agents targeting Nrf2, PtpA factors, and BACH1 inhibitors. Vitamin E and selenium can also mitigate ferroptosis and reduce cell damage through their antioxidant effects.
Epstein–Barr virus (EBV)	EBV infection activates lipid metabolism and induces ferroptosis, which may serve as a potential therapeutic strategy for EBV-associated lymphomas. It triggers ferroptosis by promoting lipid peroxidation while simultaneously upregulating Gpx4 to confer resistance to ferroptosis in NPC cells.	In the early stages of infection, EBV induces lipid metabolism and ROS production, making B cells susceptible to ferroptosis. In the later stages of infection, EBV upregulates Gpx4 to protect NPC cells from ferroptosis.	Dipyridamole (DIP) effectively inhibits EBV reactivation in B cell lines. Erastin induces significant cell death by inhibiting SLC7A11, while ML-210 induces cell death by inhibiting Gpx4.
SARS-COV-2	SARS-CoV-2 induces ferroptosis through multiple mechanisms, thereby enhancing its pathogenicity. In sinoatrial node cell lines, infection triggers ferroptosis. While in ischemic stroke, ACSL4 esterification promotes ferroptosis. The SARS-CoV-2 Orf 7b protein mediates ferroptosis via c-Myc, leading to lung injury. Additionally, ORF 3a sensitizes cells to ferroptosis through the Keap1-NRF2 axis. ORF 3a stabilizes Keap1, which promotes NRF2 degradation and relieves the inhibition of ferroptosis.	SARS-CoV-2 infection induces ferroptosis in sinoatrial node cells, damages lung cells through ferroptosis mechanisms, and exacerbates ischemic stroke via iron metabolism-related biomarkers.	Deferiprone is a chelating agent that binds to iron and is widely utilized in the treatment of iron overload. Cathepsin L (CTSL) plays a critical role in the entry of SARS-CoV-2 into host cells, and deferiprone mitigates viral entry by decreasing the RNA and protein levels of lysosomal (CTSL). Ferroptosis inhibitors (DFO, Fer-1) can counteract the effects of SARS-CoV-2 ORF3a, which acts as a positive regulator of ferroptosis in cells. Ferroptosis inhibitors such as troglitazone and Rosiglitazone have been shown to reduce viral production, including that of SARS-CoV-2.
Pseudomonas aeruginosa (*P. aeruginosa*)	*P. aeruginosa* induces ferroptosis via pLoxA and TMEM 16F, leading to lipid peroxidation and cell death in lung epithelial cells.	Ferroptosis exacerbates inflammation and damages lung tissues, particularly in cystic fibrosis patients.	Antioxidant compounds such as Idebenone, in conjunction with next-generation ferroptosis inhibitors (UAMC-3203), may effectively inhibit cell death in cystic fibrosis lung cells induced by Pseudomonas aeruginosa, as well as other forms of bacterial pneumonia, potentially leading to a significant reduction in lung inflammation.
Dengue Virus	Dengue virus alters iron metabolism, induces ferroptosis via IL-6 and CDKN2A and disrupts cellular iron homeostasis.	Ferroptosis promotes severe disease progression, enhancing vascular permeability and immune dysregulation.	Targeting IL-6-mediated pathways; modulation of ferroptosis-related genes.
Influenza Virus	Influenza Virus causes intracellular ROS accumulation and GSH depletion, triggering ferroptosis.	Ferroptosis impacts viral replication efficiency and cell survival in infected tissues.	Inhibitors like Lipstatin-1 to modulate intracellular iron levels and limit viral replication.
Herpes Simplex Virus-1 (HSV-1)	HSV-1 infection promotes the ubiquitination and degradation of KEAP1-dependent Nrf2, leading to reduced expression of downstream anti-iron deposition genes, disrupting cellular redox homeostasis, and promoting iron accumulation. HSV-1-induced ferroptosis activates the upregulation of PTGS2 and PGE2, promoting the development of encephalitis.	Ferroptosis drives neuro-pathogenesis, leading to encephalitis and other neurological complications.	Inhibition of ferroptosis to reduce neuropathological damage; targeting Nrf2 pathways. Ferroptosis inhibitor Fer-1 significantly reduces neuropathological damage and brain inflammation in HSV-1-infected mice.
*Aspergillus fumigatus*	The pathogenicity of *Aspergillus fumigatus* is contingent upon its ability to acquire iron from the host and its resistance to oxidative stress generated by the host, thereby enhancing its virulence through the action of ROS detoxifying enzymes (120). Fusarinine C plays a role in spore iron storage and intracellular iron transport in *Aspergillus fumigatus* (121).	The pathogenicity of *Aspergillus* is intricately linked to its capacity to acquire iron and its ability to respond to oxidative stress, both of which significantly enhance its survival and proliferation.	Combining targeted siderophore-mediated iron uptake with the oxidative stress response network may synergistically enhance fungal cell death (1, 3). Further investigation into targeted therapies addressing oxidative stress responses and iron metabolism could facilitate the advancement of antifungal drug development.
*Cryptococcus neoformans*	*Cryptococcus neoformans* can induce ferroptosis by elevating the levels of ferrous ions and lipid ROS. During cryptococcal infection in macrophages, significant lipid peroxidation occurs, resulting in an increased volume density of dense lipid droplets within these cells. The induction of ferroptosis by *Cryptococcus* is primarily attributed to the enhanced levels of ferrous ions and lipid ROS (122–124).	*Cryptococcus* induces ferroptosis, which enhances its pathogenicity, particularly in hosts with compromised immune systems, such as individuals infected with HIV. The ferroptosis pathway plays a crucial role in the host immune response, and disruptions in classical metabolic pathways may facilitate the development of cryptococcal meningitis.	Inhibition of the ferroptosis pathway represents a promising therapeutic strategy, which may involve the use of iron chelators and antioxidants to mitigate lipid peroxidation and ferroptosis. Treatment approaches could include the combination of antifungal agents with ferroptosis inhibitors (122).

Inhibiting ferroptosis had emerged as a promising new direction for the treatment of respiratory diseases, leading to the development of various ferroptosis inhibitors, such as deferoxamine, troglitazone, and Rosiglitazone (125–127). However, despite the promising results of these ferroptosis and iron-induced apoptosis inhibitors (such as DFO, Fer-1, and UAMC-3203) in cellular and animal models, they still face certain challenges in clinical applications (128). For instance, selectivity and targeting may pose problems, as these inhibitors could affect other physiological processes, leading to adverse reactions. Additionally, ferroptosis and iron-induced apoptosis pathways may vary across different cell types and disease states, limiting the widespread application of these molecules. Moreover, issues related to drug stability, solubility, and pharmacokinetics still need to be optimized to ensure the safety and efficacy of their clinical use.

Given the complexity of the human respiratory microbiome, the interactions between different microorganisms are intricate, and the biological effects and mechanisms of various pathogenic microorganisms differ. The precise relationship between ferroptosis and the immune system remains inadequately elucidated. Consequently, the application of ferroptosis in treating respiratory infectious diseases is still in its nascent stages. Future research should delve deeper into the relationship between ferroptosis and the respiratory microbiome to determine whether ferroptosis affects the survival and pathogenicity of other bacteria or viruses. It is also essential to identify any infection-specific ferroptosis markers, thereby providing a more robust theoretical foundation for utilizing ferroptosis as a novel approach in preventing and treating respiratory infections.
